# Supplementary material for: Modulation of biophysical properties of nucleocapsid protein in the mutant spectrum of SARS-CoV-2
Source: eLife. 2024 Jun 28;13:RP94836. doi: 10.7554/eLife.94836 (PMC11213569; doi:10.7554/eLife.94836)
Supplement: Peer review history

# Editor report: eLife assessment

**DOI:** 10.7554/eLife.94836.3.sa0

**Authors:** Mauricio Comas-Garcia

**Affiliations:** Universidad Autónoma de San Luis Potosí Mexico

**Keywords:** Convincing, Important

This **important** manuscript provides new insights into the biophysics of the SARS-CoV-2 nucleocapsid. The evidence, which relies on a **convincing** combination of genetic and biophysical data, nicely supports the conclusions.

# Referee report: Reviewer #2 (Public Review)

**DOI:** 10.7554/eLife.94836.3.sa1

This work focuses on the biochemical features of the SARS-CoV-2 Nucleocapsid (N) protein, which condenses the large viral RNA genome inside the virus and also plays other roles in the infected cell. The N protein of SARS-CoV-2 and other coronaviruses is known to contain two globular RNA-binding domains, the NTD and CTD, flanked by disordered regions. The central disordered linker is particularly well understood: it contains a long SR-rich region that is extensively phosphorylated in infected cells, followed by a leucine-rich helical segment that was shown previously by these authors to promote N protein oligomerization.

In the current work, the authors analyze 5 million viral sequence variants to assess the conservation of specific amino acids and general sequence features in the major regions of the N protein. This analysis shows that disordered regions are particularly variable but that the general hydrophobic and charge character of these regions are conserved, particularly in the SR and leucine-rich regions of the central linker. The authors then construct a series of N proteins bearing the most prevalent mutations seen in the Delta and Omicron variants, and they subject these mutant proteins to a comprehensive array of biophysical analyses (temperature sensitivity, circular dichroism, oligomerization, RNA binding, and phase separation).

The results include a number of novel findings that are worthy of further exploration. Most notable are the analyses of the previously unstudied P31L mutation of the Omicron variant. The authors use ColabFold and sedimentation analysis to suggest that this mutation promotes self-association of the disordered N-terminal region and stimulates the formation of N protein condensates. Although the affinity of this interaction is low, it seems likely that this mutation enhances viral fitness by promoting N-terminal interactions. The work also addresses the impact of another unstudied mutation, D63G, that is located on the surface of the globular NTD and has no significant effect on the properties analyzed here, raising interesting questions about how this mutation enhances viral fitness. Finally, the paper ends with studies showing that another common mutant, R203K/G204R, disrupts phase separation and might thereby alter N protein function in a way that enhances viral fitness. These provocative results set the stage for in-depth analyses of these mutations in future work.

# Referee report: Reviewer #3 (Public Review)

**DOI:** 10.7554/eLife.94836.3.sa2

Nguyen, Zhao et al. used bioinformatic analysis of mutational variants of SARS-CoV-2 Nucleocapsid (N) protein from the large genomic database of SARS-CoV-2 sequences to identify domains and regions of N where mutations are more highly represented, and computationally determined the effects of these mutations on the physicochemical properties of the protein. They found that the intrinsically disordered regions (IDRs) of N protein are more highly mutated than structured regions, and that these mutations can lead to higher variability in the physical properties of these domains. These computational predictions are compared to in vitro biophysical experiments to assess the effects of identified mutations on the thermodynamic stability, oligomeric state, particle formation, and liquid-liquid phase separation of a few exemplary mutants.

The paper is well written, easy to follow and the conclusions drawn are supported by the evidence presented. The analyses and conclusions are interesting and will be of value to virologists, cell biologists, and biophysicists studying SARS-CoV-2 function and assembly.

# Author comment: Author response

**DOI:** 10.7554/eLife.94836.3.sa3

**Authors:** Ai Nguyen, Joy Zhao, Dulguun Myagmarsuren, Sanjana Srinivasan, Di Wu, Jiji Chen, Grzegorz Piszczek, Peter Schuck

**Affiliations:** National Institutes of Health Bethesda United States; National Institutes of Health Bethesda United States; National Institutes of Health Bethesda United States; National Institutes of Health Bethesda United States; National Institutes of Health Bethesda United States; National Institutes of Health Bethesda United States; National Institutes of Health Bethesda United States; National Institutes of Health Bethesda United States

The following is the authors’ response to the original reviews.

**Public Reviews:**

**Reviewer #1 (Public Review):**

The study is highly interesting and the applied methods are target-oriented. The biophysical characterization of viable N-protein species and several representative N-protein mutants is supported by the data, including polarity, hydrophobicity, thermodynamic stability, CD spectra, particle size, and especially protein self-association. The physicochemical parameters for viable N-protein and related coronavirus are described for comparison in detail. However, the conclusion becomes less convincing that the interaction of peptides or motifs was judged by different biophysical results, with no more direct data about peptide interaction. Additionally, the manuscript could benefit from more results involving peptide interaction to support the author's opinions or make expression more accurate when concerning the interaction of motifs. Although the authors put a lot of effort into the study, there are still some questions to answer.

We thank the Reviewer for this assessment and wholeheartedly agree that there are still many questions. The main thrust of the present work was not intended to unravel the detailed mechanistic origin of all observations, but rather to juxtapose the different observations made with different viable N-protein species across the mutant spectrum, in order to get a sense of how narrowly the biophysical phenotype is confined to ensure virus viability. Such a study has become possible for the first time with the unprecedented genomic database of SARS-CoV-2. This has led to observations of non-local effects of individual mutations that are not independent and non-additive relative to the effects of other mutations, and in that sense we have inferred ‘interactions’. These might be mediated by direct contacts or indirectly through altered chain configurations. In the revised manuscript we have clarified this point.

Meanwhile, a number of documented direct physical intra-molecular and intra-dimer interactions provide a context to our study of mutation effects. The flexibility of the IDRs provides a rich variety of contacts that have been observed in molecular dynamics and single-molecule fluorescence studies (Rozycki & Boura, Biophys Chem. 2022 and Cubuk et al, Nat Communs 2021). We have previously carried out detailed hydrodynamic studies of self-association interfaces located in the leucine-rich region. More recently, NMR data just published by the Blackledge laboratory (Botova et al., bioRxiv 2024) extend the list of intra-molecular contacts with the observation of long-range intra-molecular interactions between the NTD and the CTD, NTD and the phosphorylated SR-rich region, and NTD and the previously studied leucine-rich region. The latter contacts require the C-terminal region of the linker to loop back onto the NTD, which may well introduce susceptibility to any of the linker mutations. However, detailed linker configurations are beyond the scope of the present work.

With regard to the effects of the Omicron mutations in the N-arm IDR, we have shown hydrodynamic data directly demonstrating peptide self-association, and we are currently working on a more detailed functional follow-up study which we hope to communicate soon.

**Reviewer #2 (Public Review):**

Summary:This work focuses on the biochemical features of the SARS-CoV-2 Nucleocapsid (N)protein, which condenses the large viral RNA genome inside the virus and also plays other roles in the infected cell. The N protein of SARS-CoV-2 and other coronaviruses is known to contain two globular RNA-binding domains, the NTD and CTD, flanked by disordered regions. The central disordered linker is particularly well understood: it contains a long SR-rich region that is extensively phosphorylated in infected cells, followed by a leucine-rich helical segment that was shown previously by these authors to promote N protein oligomerization.

In the current work, the authors analyze 5 million viral sequence variants to assess the conservation of specific amino acids and general sequence features in the major regions of the N protein. This analysis shows that disordered regions are particularly variable but that the general hydrophobic and charge character of these regions are conserved, particularly in the SR and leucine-rich regions of the central linker. The authors then construct a series of N proteins bearing the most prevalent mutations seen in the Delta and Omicron variants, and they subject these mutant proteins to a comprehensive array of biophysical analyses (temperature sensitivity, circular dichroism, oligomerization, RNA binding, and phase separation).

Strengths:

The results include a number of novel findings that are worthy of further exploration. Most notable are the analyses of the previously unstudied P31L mutation of the Omicron variant. The authors use ColabFold and sedimentation analysis to suggest that this mutation promotes the self-association of the disordered N-terminal region and stimulates the formation of N protein condensates. Although the affinity of this interaction is low, it seems likely that this mutation enhances viral fitness by promoting N-terminal interactions. The work also addresses the impact of another unstudied mutation, D63G, that is located on the surface of the globular NTD and has no significant effect on the properties analyzed here, raising interesting questions about how this mutation enhances viral fitness. Finally, the paper ends with studies showing that another common mutant, R203K/G204R,disrupts phase separation and might thereby alter N protein function in a way that enhances viral fitness.

Thank you for highlighting the strengths of our paper.

Weaknesses:

In general, the results in the paper confirm previous ideas about the role of N protein regions. The key novelty of the paper lies in the identification of point mutations, notablyP13L, that suggest previously unsuspected functions of the N-terminal disordered region in protein oligomerization. The paper would benefit from further exploration of these possibilities.

We agree that the bioinformatic results confirm previous ideas about the role of the N protein regions. However, we believe our results go beyond the previous thinking in a crucial aspect, which is that we examine the full (so far known) mutant spectrum of N-protein. Properties previously inferred from the inspection of single consensus sequences can be misleading because of the quasispecies nature of RNA viruses. By considering the mutant spectrum we can obtain a sense for how significant differences in the physicochemical properties of the different regions are, and how much variation is possible without jeopardizing essential protein functions.

With regard to the N-arm IDR mutations we believe this deserves a separate study focusing on the apparent N-arm function. Our rationale for presenting some initial N-arm results in the current paper was to highlight how the variability of N-protein species in the mutant spectrum can even include differences in the type and number of protein self-association interfaces.

**Reviewer #3 (Public Review):**

Nguyen, Zhao, et al. used bioinformatic analysis of mutational variants of SARS-CoV-2Nucleocapsid (N) protein from the large genomic database of SARS-CoV-2 sequences to identify domains and regions of N where mutations are more highly represented and computationally determined the effects of these mutations on the physicochemical properties of the protein. They found that the intrinsically disordered regions (IDRs) of N protein are more highly mutated than structured regions and that these mutations can lead to higher variability in the physical properties of these domains. These computational predictions are compared to in vitro biophysical experiments to assess the effects of identified mutations on the thermodynamic stability, oligomeric state, particle formation, and liquid-liquid phase separation of a few exemplary mutants.

The paper is well-written and easy to follow, and the conclusions drawn are supported by the evidence presented. The analyses and conclusions are interesting and will be of value to virologists, cell biologists, and biophysicists studying SARS-CoV-2 function and assembly. It would be nice if some further extrapolation or comments could be made regarding the effects of the observed mutations on the in vivo behavior and properties of the virus, but I appreciate that this is much higher-order than could be addressed with the approaches employed here.

We thank the Reviewer for this positive assessment. With regard to the possible in vivo behavior of mutant species, we agree that this would require additional data beyond the scope of the present work.

However, for the N:G215C mutant we can point to a very recent preprint by Kubinski et al. (bioRxiv 2024) that describes reverse genetics experiments where the isolated N:G215C mutation caused altered in vivo pathology, enhanced viral replication, and altered virion morphology. We have cited this work in the revised manuscript.

As mentioned above, for the P13L mutation we hope to communicate a more detailed follow-up study that will allow us to extrapolate on its in vivo behavior.

**Recommendations For The Authors:**

**Reviewer #1:**

(1) Given the structure organization of N-protein in Figure 1, the authors should explain why linker region 180-247 is different from linker (175-247) mentioned in the first result.

We thank the reviewer for bringing up this point, which we agree deserves clarification. While often the NTD has been assigned a C-terminal limit of 180 (e.g., in the NMR structure by Dinesh et al, Plos Pathogens 2020), the last several residues in the NTD are already disordered and contain the S176/R177 pair and therefore may be ascribed to the beginning of the SR-rich portion of the linker. In order not to artificially truncate functional sequences of either NTD or linker, we have decided to allow the designations of the NTD and linker regions to overlap. We believe this is conservative in that possible NTD or linker properties extending into this transition region will be preserved. In order to explain this in the manuscript, we have modified Figure 1 and inserted a brief sentence “(Due to ambiguity in delineation between NTD and linker, designations overlapping in 175-180 were used to avoid artificial truncation and permit conservative evaluation of the properties of each domain.)”.

(2) Please specify the "physicochemical requirements" in the fourth paragraph of the first result, and its physicochemical meaning and references.

Thank you for pointing this out; we agree this was not well expressed. We have rephrased this (including new references) to “…we find that hydrophobicity is uniformly high and polarity correspondingly low in the folded NTD and CTD domains, which is consistent with the expectation that folded structures are stabilized by buried hydrophobic residues (Eisenberg and McLachlan, 1986; Kauzmann, 1959)”.

(3) The authors should clarify the biological meaning of the net charge and phosphorylation charge in the first result, just like the description in the results of polarity and hydrophobicity.

We agree this will improve readability, and have inserted an introductory sentence to the study of charges in the mutant spectrum: “Charges in proteins can control multiple properties related to electrostatic interactions, from functions of active sites to protein solubility, protein interactions, and conformational ensembles in IDRs (Garcia-Viloca et al., 2004; Gerstein and Chothia, 1996; Gitlin et al., 2006; Mao et al., 2010).”.

(4) The authors should clarify the calculation method and meaning of the column "occurs in % of all genomes" in Table 2.

We have inserted a footnote specifying that this is the “Percentage of all sequenced genomes carrying the specific mutation.”.

(5) Please specify what information or conclusion we can get for the shift of the intrinsic fluorescent spectrum of N: D63G in the third result paragraph 2.

We have rephrased the second sentence of this paragraph to “The presence of the N:D63G mutation in the NTD is highlighted in the shift of the intrinsic fluorescence quantum yield of this mutant in comparison to Nref ”. It confirms the structural prediction, which positions D63G at the protein surface near the NA binding site, and sets up the question whether this obligatory mutation of Delta-variant N-protein affects NA binding and thereby possibly assembly. Unexpectedly, we did not find any impact of the D63G mutation on NA binding, although we observed a modest impact on temperature-dependent particle formation by DLS.

(6) The conclusion, "some epistatic interaction between mutation of the linker and N-arm" in the third result paragraph 4, is over-interpreted from the result of the CD spectra because they didn't detect peptide interaction between mutation of the linker and N-arm.

Thank you for raising this point. We did not mean to make a strong conclusion here, and have now deleted this statement.

(7) The parallel assay for N: G215C and Nδ in SV-AUC experiments is recommended to be conducted with other groups to avoid experimental error.

I believe this may be a misunderstanding: Indeed we had carried out SV-AUC experiments for all the mutants, as shown in Figure 5A. However, since all but the N:G215C and Nδ formed only dimers as the reference protein, we did not comment on these in the results text. We have rectified this omission in the revision by inserting the sentence: “…The same behavior is observed for N:D63G, No, N:R203K/G204R, as well as N:P13L/Δ31-33 at low micromolar concentrations (Figure 5A). By contrast, the G215C mutation promotes the formation of higher oligomers…”

With regard to experimental error, SV-AUC is an absolute method based on first principles and we have maintained our instruments by performing regular calibrations, using methods developed by us and colleagues at NIST, as described in the literature (Anal Biochem 2013, PLOS ONE 2018, Eur. Biophys. J. 2021). Previously we have critically examined the accuracy of s-values by SV-AUC before and after calibration in a large multi-laboratory study (PLOS ONE 2015), and found that the accuracy of s-values is ~1%. This allows detailed comparisons of results from different runs and different points in time. To alleviate any concerns we have now mentioned our calibration methods in the methods section.

(8) The authors did not test the function of Nδ R203M mutation, so they should not mention about it like in the third result paragraph 5, which is over-interpreted from result 5A.

We accept the criticism that we have not yet examined the R203M mutation in isolation. However, we believe some speculation is in order: Nδ consists of D63G, R203M, G215C, and D377Y, of which D63G is unlikely to impact oligomeric state based on our data of N:D63G. It is therefore reasonable to assume that R203M and/or D377Y interfere with the observed promotion of oligomerization that we have observed with N:G215C. In previous work, we have traced the 215C-incuded oligomerization to the transient helix in the leucine-rich region of the linker 215-235 (Science Advances, 2023), Since 377Y is quite far away, the more proximal 203M appears to be the most plausible origin of the modulation of dimerization.

In the revision we have more clearly outlined this speculation: “ Of the three additional mutations of Nδ relative to N:G215C, we speculate that D63G does not impact dimerization (as in N:D63G, Figure 5A), and that therefore either the distant D377Y and/or R203M might cause this reduction of helicity and oligomerization relative to N:G215C, noting that R203M is proximal to the L-rich region (215-235) reshaped by 215C. ”. Later we refer to this as “any potential inhibitory role suspected of the R203M mutation on self-association…”.

(9) The description of LLPS formation lacks reference in the third result paragraph 6.

Thank you. To improve the transition to this new paragraph in the results, we have inserted “As outlined in the introduction, …” and repeated the 8 references to the fact that N-protein undergoes LLPS. The two additional, separate references refer to just those published studies that examined the temperature-dependence of LLPS, which I believe is now clearer.

(10) The authors did not test the interaction between the N-arm IDR mutation and linker IDR, it is not exponible that interaction promoted particle formation of No in the third result paragraph 8, which is over-interpreted from result 5B.

We thank the Reviewer for raising this point. In fact, we did not want to imply a direct physical interaction (in terms of binding) between the N-arm IDR mutation and that in the linker. But clearly there are non-additive effects in particle formation since P13L/Δ31-33 inhibits slightly and R203K/G204R inhibits almost completely, whereas the combination of the two (constituting No) promotes particle formation. We have rephrased this to “alter the effect of”, avoiding the term “interact with” not to suggest a picture of direct binding and invoke instead the idea of epistatic interactions.

(11) In the third result paragraph 9, why did the authors choose to examine the role of the N-arm mutations of the Omicron variants in greater detail? This reason should be added to the manuscript.

Thank you for this suggestion. Naturally, we were curious how the defining N-arm mutations of Omicron variants could impact particle formation. Even though no obvious enhancement of self-association by either Omicron N-arm or linker mutations was observed at low micromolar concentrations in SV-AUC (Figure 5A), we knew from experience with the study of the leucine-rich transient helix in the linker IDR that even weak interfaces with mM Kd can be highly relevant in the context of multivalent assemblies (Science Advances, 2023). Therefore we followed the same roadmap and focused on IDR peptides with the goal to study them at higher concentrations that might reveal weak interactions.

We have described this motivation as follows: “We were curious whether IDR mutations might alter particle formation through modulation of existing or introduction of new protein-protein interfaces. We focused on Omicron mutations as these are obligatory an all currently circulating strains, and specifically on N-arm mutations, which have recently been implicated in altered intramolecular interactions with NA-occupied NTD (Cubuk et al., 2023). Even though SV-AUC showed no indication of self-association of N:P13L/Δ31-33 at low micromolar concentrations, weak interactions with Kd > mM would not be detectable under these conditions yet could be highly relevant in the context of multi-valent complexes (Zhao et al., 2024). Following the roadmap used previously for the study of the weak self-association of the leucine-rich linker IDR (Zhao et al., 2023), we restricted the protein to the N-arm peptide such that it can be studied at much higher concentrations. To this end, we …”

(12) Why were different proteins dissolved in either high-salt buffer or low-salt buffer for biophysical experiments? Did this affect the experimental results? Explanations and evidence are required.

We appreciate this is an important point. Unfortunately, for practical reasons of available sample concentrations and quantities, it was not always possible to dialyze protein into both buffers. For example, the DSF data in Figure 4B show all proteins in low-salt buffer except N:R203K/G204R, which is in high-salt buffer. We had previously reported the absence of changes in Ti in DSF for Nref in the two buffers, which we have documented better in the revised manuscript by providing an additional Supplementary Figure S7: “As a buffer control, the difference in Ti for Nref in LS and HS buffer was measured and found to be within error of data acquisition (Supplementary Figure S7A).” This new Supplementary Figure provides an overlay of low-salt and high-salt DSF data for Nref, N:D63G, and No, which have variations in the Ti values for different buffers on the order of 0.1 °C. This is comparable to the precision of the measurement, and significantly smaller than the changes in Ti values between the different mutant protein species. Finally, we note that the one species for which we were unable to collect DSF data in low-salt buffer, N:R203K/G204R, was unremarkable relative to Nref, No, and N:P13L/Δ31-33.

In the case of CD, the only species for which we could not collect spectra in low-salt buffer was No. Again, this spectrum was similar to the group including Nref, along with N:P13L/Δ31-33, and N:D63G. In the results we interpreted significant differences from Nref for N:G215C and N:R203K/G204R.

Similarly, SV-AUC experiments were carried out in high-salt buffer, except Nref, Nδ , and N:G215C. In this case, we could observe a ≈ 5% difference in s-value for the same protein in different buffers, but the magnitude of this change is negligible compared to the ≈ 60-90% increase observed for altered oligomeric states. To clarify this we have inserted a sentence “Proteins for self-association studies were in buffer HS, except Nref, Nδ , and N:G215C were in LS, the latter causing a ≈5% increase in s-value (Supplementary Figure S7B).”, with the new Supplementary Figure S7B showing a comparison of sedimentation coefficient distributions of Nref and N:D63G in low- and high-salt buffers. Whether the small differences in s-values are indeed significant and reflective of salt-dependent conformational ensembles of IDRs will require a more detailed follow-up study, but is outside the scope of the present work.

All other experiments were carried out with uniform buffer conditions for all protein species.

(13) DLS data of N from other research suggests oligomers beyond dimer. Please address this discrepancy.

Unfortunately several previous studies in the literature did not recognize the importance of eliminating nucleic acid contaminations in the N-protein preparations, and/or did not succeed in completely removing nucleic acid from the protein. We and others have repeatedly commented on this issue. For example, Tarczewska et al (IJBM 188 (2021) 391-403) clearly demonstrate this in much detail in a study dedicated to this problem.

The clarify this point we have included a sentence in the paragraph describing the protein preparation “…the ratio of absorbance at 260 nm and 280 nm of ~0.50-0.55 confirmed absence of nucleic acid. The latter is important to eliminate higher order N-protein oligomers induced by nucleic acid binding (Carlson et al., 2020; Tarczewska et al., 2021; Zhao et al., 2021)” .

In order to strengthen the statement in the Results that the ancestral N-protein is dimeric we have added additional references from other labs that have carried out detailed biophysical analyses: “As reported previously, the ancestral N-protein at micromolar concentrations in NA-free form is a tightly linked dimer sedimenting at ≈4 S , without significant populations of higher oligomers (Forsythe et al., 2021; Ribeiro-Filho et al., 2022; Tarczewska et al., 2021; Zhao et al., 2022, 2021).”

**Reviewer #2:**

The key novel finding of the work lies in the evidence that P31L promotes N-terminal interactions. The paper would be strengthened by additional studies of the impact of P31Lon the oligomerization of full-length N protein. The sedimentation analysis in Fig 6 shows that high concentrations of the N arm alone self-associate, while the analysis in Fig 5 argues that P31L does not have an effect on the oligomerization of the full-length protein. Perhaps there are specific conditions or mutation combinations that would provide evidence that P31L has an effect on protein behavior that might explain the prevalence of this mutation.

We agree that the finding of P13L promoting N-terminal interactions is of great interest, and we thank the Reviewer for the suggestion to examine cross-correlations of N-arm mutations with other mutations as a tool to study its function and relevance.

The observation of self-association in Figure 6 at high concentrations is not necessarily at odds with the absence of self-association at 100fold lower concentrations. Rather, it seems to show that the interaction mediated by the N-terminal mutation P13L is weak with an effective Kd in the mM range. It will likely not be possible to reach sufficiently high protein concentrations with the full-length protein to visualize the oligomerization of N-terminal IDR. But even if it was possible to concentrate the protein enough, very likely other assembly processes would take place, including LLPS, obscuring potential P13L interfaces. Nonetheless we believe the protein-protein interface created by the N-arm IDR is highly relevant in the context of multi-valent complexes, where entropic co-localization enhances the effective N-arm IDR concentration that then can provide additional binding energy and strengthen the assembly of multi-protein complexes.

We are currently pursuing further experiments examining the properties and relevance of the N-arm mutations and intend to publish this in a separate study, not to distract from the thrust of the current work exploring of the extent of the biophysical phenotype space.

The R203K/G204R mutations have a surprising impact on LLPS in Figure 7: it is not clear how such limited mutations would alter the many nonspecific, multivalent interactions that presumably lead to phase separation. The paper would benefit from a more extensive analysis of LLPS in this mutant and in the P31L mutant, perhaps by performing the analysis at various protein concentrations and times.

Following this recommendation we have expanded the study of LLPS of Figure 7 by comparison of two different time points for Nref, N:R203K/G204R, and N:P13L in a new Supplementary Figure S6. We have also quantified the droplet distributions as shown in the new Supplementary Figure S5. Both clearly confirm the strong inhibitory effect of the R203K/G204R mutation on LLPS under our experimental conditions. What this shows is not that this protein could not undergo LLPS per se, but that the phase boundaries have shifted such that under the experimental conditions we applied LLPS does not occur yet. (In this context it is interesting to note that ≈50,000 genomes in the GISAID database have R203K/G204R as the sole N-protein mutation, without impact on viral viability.)

That individual point-mutations in IDRs can have significant impact on LLPS has been observed previously for several other proteins. Examples include SPOP [Bouchard et al., Mol Cell 72 (2018) 19-36.e8], SHP2 [Zhu et al., Cell 183 (2020) 490-502.e18], FUS [Niaki et al., Mol Cell 77 (2020) 82-94.e4], and CAPRIN1 [Kim et al., PNAS 118 (2021) 1-11]. The latter work applies NMR and reveals that promotion of LLPS is not uniform but centered in hot-spot residues of CAPRIN1.

While the precise molecular mechanism for LLPS of the N-protein is unclear, we can speculate how the effect of 203K/204R might be amplified. As shown by the coarse-grained MD simulations from Rozycki & Boura (Biophys. Chem. 2022), the linker IDR is highly flexible and the 203/204 residues make transient contacts to other residues throughout the linker as well as to distinct sites on the NTD. Furthermore, recent NMR data from the Blackledge lab (Botova et al., bioRxiv 2024, doi:10.1101/2024.02.22.579423) have revealed intra-molecular interactions, including a state where the L-rich (C-terminal) portion of the linker IDR interacts with a site on the distant NTD. (We have included a reference to this preprint in the discussion.) This intra-molecular contact observed in NMR must cause significant chain compaction and may thereby modulate the accessibility of portions of the linker IDR available to inter-molecular interactions contributing to LLPS. The residues 203/204 are in the middle between the SR-rich and L-rich region where bending of the chain must occur to allow for the intra-molecular contacts. The 203K/204R mutation may alter the dynamics or population of this intra-molecular bound state, especially considering the introduction of a bulky positively charged R replacing G204.

In summary, considering the dynamics of intra-molecular contacts and considering precedent of several other disordered proteins, we believe it is not unreasonable that the local mutation in the IDR R203K/G204R may cause a significant shift in LLPS phase boundaries. We note that this mutant also shows a very distinct behavior in the temperature-dependent DLS, entirely lacking particle formation below 70 °C. This observation seems consistent with altered inter-molecular interactions.

**Reviewer #3:**

I have only a few minor specific comments:

(1) Page 4, last paragraph - typo: "The large number of structural and non-structural N-protein functions poses the question of how they are conserved...". This either needs a colon or to be changed to "... poses the question of how they are conserved...".

Thank you – we have changed this sentence accordingly.

(2) Page 7, 2nd and 3rd paragraphs of "Physicochemical properties" section: why is Figure2B discussed before Figure 2A?

Initially when we present the results of polarity and hydrophobicity we refer more generally to Figure 2, as the two properties are so closely related. Later, in the section on related coronaviruses we do refer once more to Figure 2. Here we begin this section by discussing Figure 2B since in this plot the symbols for the different viruses are most recognizable.

(3) Page 11, lines 1-2: "Since this is a tell-tale of weak protein..." -> "tell-tale sign of ...".

We thank the reviewer for pointing this out and have fixed this sentence.

(4) Further down in the same paragraph, the meaning of "SV-AUC" should be spelled out at its first use.

We have double checked that SV-AUC is spelled out at its first use.

(5) Figures 1 and 2. Is there a good reason that the color scheme for the IDRs (magenta and cyan) is so close to the color scheme for the identifying mutations of Omicron and Delta (magenta and blue)? This initially led me to try to search for some connection, and it remains unclear to me if there is.

We apologize for this confusion. This was indeed a poor color choice, and we have rectified this in the revised manuscript by changing the colors of the identifying mutations of Omicron and Delta to dashed green and dotted red, respectively, so that there is no connection to the shading of the IDRs. Thank you very much for pointing this out!

(6) Figure 1: The physical limits of the subdomains, e.g. SR-rich, L-rich, C-arm1, and N3 could be more clearly delineated with lines, or some other visual representation.

Once more, we thank the reviewer for pointing this out. We have revised Figure 1 to indicate the limits between these subdomains.

(7) Figures 4, 5, and 6: are there any kind of error bars or confidence intervals on these measurements?

We appreciate this concern and have addressed it in different ways for the different methods.

For the spectra of intrinsic fluorescence in Figure 4A, we have now plotted an overlay of three acquired spectra, from which the experimental error as a function of wavelength may be assessed. It is clear that the differences between Nref and N:D63G are far greater than the measurement error.

With regard to DSF, we have provide an error estimate of 0.3 °C for the Ti-values, a value that we have revised from the previously reported errors of sequential replicates to now include Ti variation observed with different preparations of the same protein over long time periods.

For CD spectra we have included a new Supplementary Figure S3 that shows standard deviations of triplicate measurements as a function of wavelength. Since an overlay including errors for all species would be too crowded, we have created separate plots for all species in comparison with Nref. (On this occasion we discovered a 3% error in the magnitude of the Nref spectrum due to previously incorrect conversion to MRE, which we have now fixed.)

In SV-AUC, for data with typical signal-noise ratio, the statistical error is very small due to the large number (> 104 ) of raw data points included in the calculation of each c(s) trace, which each data point carrying a statistical error that is usually better than 1%. Therefore, the dominant error is systematic. In the past we have carried out large studies quantifying the accuracy of the major peaks of the sedimentation coefficient distributions, and found they are typically ≈1% in s-value and 1-2% for relative peak areas. In the AUC methods section we have now included the sentence “Typical accuracy of c(s) peaks are on the order of ≈1% for peak s-values and ≈1-2% for relative peak areas (Zhao et al., 2015).”

Finally, for the temperature-dependent DLS data we have to resort to the scatter in the temperature-dependent Rh-values. The calculated Rh-values can exhibit fluctuations once particles start to form and the distribution becomes highly polydisperse. As is characteristic for DLS under those conditions, individual Rh-values can be dominated by adventitious diffusion of few large particles into the laser focal spot. Although customarily autocorrelation functions can be filtered out through software filters (e.g., setting baseline and amplitude thresholds), this still presents the largest source of error in the Rh-values. These are systematic for the individual autocorrelation functions. We believe that the variation of Rh-values at similar temperatures outside the transition region provides a reasonable estimate for the experimental error.

(8) Figure 7: My most major comment. It would be good to somehow quantify the differences between these images. The claim is made that the LLPS droplets are different sizes, or for the P13L/\Delta31-33 variant that droplets are coalescing or changing shape over time. It would be good to quantify this rather than rely on eyeballing the pictures.

We are grateful to the Reviewer for this suggestion. As mentioned above, to improve the LLPS analysis we have now carried out segmentation of the images in Figure 7 to quantify the droplet numbers and areas. Histograms and statistical analyses are now provided in the new Supplementary Figure S5. In addition, we have added a comparison of the droplet numbers and sizes at two time-points for Nref, N:R203K/G204R, in addition to the previously shown N:P13L/Δ31-33, provided in the new Supplementary Figure S6. The results corroborate the previous conclusions, and depict how droplets in the N:P13L/Δ31-33 merge and grow in area more strongly than those from Nref.
